# Annotations

**Published:** 1902-04-19

**Authors:** 


### ANNOTATIONS.

### Medical Qualifications.

The evils connected with the present system of handing over the work of examining candidates for medical qualifications to 19 separate and more or less independent bodies are again brought into prominence, on the one hand by the threatened disruption of the Victoria University and the consequent still further increase in the number of examining bodies which would result were three separate degree-giving Universities to rise from the ashes of the one destroyed, and on the other hand by the recently proclaimed intention of some of the existing bodies to act independently of the General Medical Council in regard to the course of study which they will require from their candidates. We seem indeed to have before us every prospect of an era of open competition between the various dealers in medical qualifications, a disastrous condition of affairs which is sure to give much force to the demand, so often made, for a single State Examination and a single State Licence to practise. There are many things in medicine which are allowed to continue merely because they exist, because they are in accordance with traditions, and because they are said to "work well." If, however, in addition to all the theoretical objections which may be brought against the present anomalous and complicated system of medical qualification, it can also be shown that it does not work, its doom is sealed.

### The Afforestation of Water-Bearing Areas.

As a country becomes more populous water supply becomes a more and more urgent problem, and many large towns have spent enormous sums upon gathering grounds, which they have depopulated and left deserted lest the purity of the water draining from them should be imperilled. The permanent appropriation of large portions of the country for the single purpose of acting as catchment areas becomes then a serious national question, and much interest attaches to an article by W. B. Crump, M.A., and W. G. Smith, B.Sc., Ph.D., in the *Halifax Naturalist* for this month, in which it is urged that both as a means of improving the land for its purpose as a gathering ground and with the object of getting something besides mere water from the land, the catchment areas should be carefully planted so as to grow useful forms of timber. It appears that for water purposes alone the Halifax Corporation have become owners of thousands of acres of land which they are anxious to preserve for all time unpeopled and uncultivated. The same is probably the case with other towns. Farms have passed out of cultivation and will revert to moorland, and it is maintained that by afforestation this land could not only be rendered productive, but that the presence of trees would actually improve it as a source of an abundant and regular supply of pure water. Anything which offers to diminish the enormous cost and the apparent wastefulness of devoting large tracts of land to water production alone is a matter of great sanitary importance, for no one can regard with satisfaction the plan which, unfortunately, some towns have to put up with, of using the drainage from cultivated and inhabited areas as the sources of their water supply.

### The Cancer Investigation.

The publication of the complete scheme for the systematic investigation of cancer, which has now received the sanction of the Royal Colleges, draws attention afresh to this important pathological problem. The long, and so far as practical utility is concerned, the somewhat ominous list of dignified officials who are to preside over the inquiry—that is, when the £100,000 has been provided, which is not yet—will, no doubt, produce in the public mind a full assurance that in face of such a galaxy of talent cancer will soon be brought to bay and forced to give up its long-hidden secret. We confess we have no such hopes. It is not by endless committees and carefully-worded resolutions that inquiries of this sort can be rendered fruitful. Individual work, and we may add individual imagination, will be required, and it is fair to doubt whether the ponderous mass of official machinery with which the published scheme is weighted will not greatly interfere with its practical utility. No one can pretend that the fact of being President for the time-being of one of the Royal Colleges, or being a member of the laboratories committee, or being nominated by the Royal or other society, gives a man the slightest right to speak authoritatively on such a special subject. What the scheme may be expected to do, however, will be to ensure the respectability of the methods and the *bonâ fides* of the results. Into all such inquiries as this, hydra-headed quackery tends to intrude at every turn, and the utility of the scheme lies in the respectability of the doorkeepers which it provides. But herein also lies its danger. At present we know nothing with any approach to certainty. Yet, even to-morrow, someone may strike the trail; and then the question will arise, "Will these doorkeepers recognise the discoverer?"

### Portsmouth Hospital.

A special report, which has been presented to the committee of management of the Portsmouth Hospital shows that the arrangements in the out-patient department of that institution are far from what they ought to be. Complaint is more particularly made of the lack of decency in the surgical department. It is stated that the dressings are done in the "casualty wards," where men and women are congregated together, being only separated by a screen which is described as "ragged and inadequate." It is further pointed out that the women, in order to reach their department, have to pass men whose clothes are disarranged and whose wounds are exposed, and that when the morning dressings are being done no curtain is used, the patients being ranged round the room "indiscriminately, no regard being paid to age or sex." The committee, on receiving this report, appear to have put off doing anything until the next monthly meeting, but there are evidently a good many people in Portsmouth who think that some immediate action ought to have been taken. The whole business seems to depend upon lack of proper organisation. Anyone blessed with a reasonable amount of authority could, in a very short time, reduce this chaos into order. But Portsmouth is not the only hospital where a strong hand in the out-patient department might do much for decency.

